# 

**Published:** 1854-12

**Authors:** 


### CONTENTS.

PAGE.

ART. I.—ADDRESS AT THE OPENING OF THE SESSION FOR 1854-55 IN THE MEDICAL DEPARTMENT OF THE UNIVERSITY OF LOUISVILLE. By AUSTIN FLINT, M. D. ....409
ART. II.—M. DEVERGIE ON BATHS, ETC. TRANSLATED BY PROF. FLINT...426
ART. III.—CASES AND OBSERVATIONS. By W. H. COBB, M. D. ....434
ART. IV.—GUM MEZQUITE AS A SUBSTITUTE FOR GUM ARABIC. By GEORGE G. SHUMARD, M. D. ....437
ART. V.—A CASE OF SALIVARY FISTULA. By F. H. BADGER, M. D. ....439
ART. VI.—FOREIGN CORRESPONDENGE. By E. WILLIAMS, M. D. ....441

BIBLIOGRAPHICAL NOTICES:

ART. VII.—THE PRINCIPAL FORMS OF THE SKELETON AND OF THE TEETH. By PROF. R. OWEN, F. R. S., &c. ....459
ART. VIII.—A CLINICAL INTRODUCTION TO THE PRACTICE OF AUSCULTATION. By H. M. HUGHES, M. D. ....462
ART. IX.—THE TRANSACTIONS OF THE AMERICAN MEDICAL ASSOCIATION. ....464
ART. X.—NOTES OF M. BERNARD'S LECTURES ON THE BLOOD. By WALTER F. ATLEE, M. D. ....466

EDITORIAL DEPARTMENT: PAGE.

Close of the Volume. A Fatal Case of Intermittent Fever Treated Homœopathically. ....467
The Case of Dr. Beale ....468
Professor Flint's Introductory ....469
Dr. Drake's Work. Gross on Foreign Bodies in the Air-Passages. New Publications ....470

RECORD OF MEDICAL SCIENCE: PAGE.

Discovery of Viviparous Fish in Louisiana ....473
Bilateral Operation in the Female for Calculus ....476
Improvements in Surgery ....477
Membranous Croup ....479
Mr. Spencer Wells. A Case of Poisoning. ....480
Local Anæsthesia ....481
Simple and Ozonized Oil of Turpentine ....482
The Employment of External Heat and Cold. Strychnine ....483
Spongy Bougies. Professor Simpson ....484
Lupus. Biniodide of Mercury. Dippel's Animal Oil. Leucorrhœa. Albugo ....485
Laryngitis. Nitrate of Silver. Poisoning by Lead. Chronic Rheumatism ....486
Tetanus. Chloroform. Tinea. Sulph. Acid. Fibrous Tumour...487
Epilepsy. Oxide of Zinc. Epilepsy. Atropine. Subnitrate of Bismuth. Maganese in Chlorosis ....488

